# *Dec1* Deficiency Ameliorates Pulmonary Fibrosis Through the PI3K/AKT/GSK-3β/β-Catenin Integrated Signaling Pathway

**DOI:** 10.3389/fphar.2022.829673

**Published:** 2022-03-09

**Authors:** Xingxing Hu, Menglin Zou, Lan Ni, Mingyang Zhang, Weishuai Zheng, Bing Liu, Zhenshun Cheng

**Affiliations:** ^1^ Department of Respiratory and Critical Care Medicine, Zhongnan Hospital of Wuhan University, Wuhan, China; ^2^ Wuhan Research Center for Infectious Diseases and Cancer, Chinese Academy of Medical Sciences, Wuhan, China

**Keywords:** pulmonary fibrosis, differentiated embryonic chondrocyte expressed gene 1, epithelial-mesenchymal transition, PI3K/AKT/GSK-3β/β-catenin integrated signaling pathway, bleomycin, TGF-β1

## Abstract

Tissue remodeling/fibrosis is a main feature of idiopathic pulmonary fibrosis (IPF), which results in the replacement of normal lung parenchyma with a collagen-rich extracellular matrix produced by fibroblasts and myofibroblasts. Epithelial-mesenchymal transition (EMT) in type 2 lung epithelial cells is a key process in IPF, which leads to fibroblasts and myofibroblasts accumulation and excessive collagen deposition. DEC1, a structurally distinct class of basic helix-loop-helix proteins, is associated with EMT in cancer. However, the functional role of DEC1 in pulmonary fibrosis (PF) remains elusive. Herein, we aimed to explore DEC1 expression in IPF and bleomycin (BLM)-induced PF in mice and the mechanisms underlying the fibrogenic effect of DEC1 in PF *in vivo* and *in vitro* by *Dec1*-knockout (*Dec1* ^−/−^) mice, knockdown and overexpression of *DEC1* in alveolar epithelial cells (A549 cells). We found that the expression of DEC1 was increased in IPF and BLM-injured mice. More importantly, *Dec1* ^−/−^ mice had reduced PF after BLM challenge. Additionally, DEC1 deficiency relieved EMT development and repressed the PI3K/AKT/GSK-3β/β-catenin integrated signaling pathway in mice and in A549 cells, whereas *DEC1* overexpression *in vitro* had converse effects. Moreover, the PI3K/AKT and Wnt/β-catenin signaling inhibitors, LY294002 and XAV-939, ameliorated BLM-meditated PF *in vivo* and relieved EMT *in vivo* and *in vitro*. These pathways are interconnected by the GSK-3β phosphorylation status. Our findings indicated that during PF progression, DEC1 played a key role in EMT via the PI3K/AKT/GSK-3β/β-catenin integrated signaling pathway. Consequently, targeting DEC1 may be a potential novel therapeutic approach for IPF.

## 1 Introduction

Idiopathic pulmonary fibrosis (IPF), the most common type of idiopathic interstitial pneumonia (IIP), is a chronic, irreversible and typically fatal fibrotic lung disease, which is characterized by overproduction and disorganized deposition of extracellular matrix (ECM) proteins, together with abnormal proliferation of mesenchymal cells, ultimately leading to distortion of pulmonary architecture and impairment of pulmonary functions (Okamoto et al., 2011; Wolters et al., 2018). The incidence and prevalence of IPF reportedly range from 2 to 30 cases per 100,000 person-years and 10–60 cases per 100,000 people, respectively. The disease has a grave prognosis with an estimated median survival time of 3–5 years following diagnosis if untreated (Martinez et al., 2017). Currently, only two drugs, nintedanib and pirfenidone, are FDA-approved for the treatment of IPF, but they only reduce the rate of lung function decline, and they may have several serious side effects (Kasam et al., 2020). At present, the pathogenesis of IPF is poorly understood. Therefore, it is necessary to investigate the precise mechanisms underlying IPF and identify new antifibrotic therapeutic approaches.

In recent years, the proposed mechanisms involved in IPF have been reported to include epithelial cell dysfunction, fibroblast proliferation and excessive ECM production (Inoue et al., 2020). Dysregulation of type 2 alveolar epithelial cells (AEC2s) is thought to be central in fibrogenesis in IPF (Richeldi et al., 2017). Fibroblasts and myofibroblasts, which express the contractile protein, α-smooth muscle actin (α-SMA), and produce excessive ECM, are the key sources of ECM and are implicated in the pathogenesis of pulmonary fibrosis (PF). In the lung, myofibroblasts are mainly derived from activated lung fibroblasts. Additionally, it has been reported that epithelial microinjuries (of a yet unknown cause) trigger abnormal epithelial-mesenchymal interactions and pathogenesis of IPF(Fernandez and Eickelberg, 2012). More importantly, emerging evidence suggests that epithelial-mesenchymal transition (EMT) of alveolar epithelial cells contributes to the cellular origin of fibroblasts and myofibroblasts accumulation, ultimately resulting in the development of PF (Chen et al., 2013; Goldmann et al., 2018; Hill et al., 2019). EMT is a multifunctional process in which epithelial cells lose the epithelial phenotype and acquire the mesenchymal phenotype, accompanied by down-regulation of epithelial cell marker (e.g., E-cadherin) and upregulation of mesenchymal cell marker (e.g., N-cadherin and Vimentin), thereby promoting PF development (Thiery et al., 2009; Peng et al., 2020). A growing body of research has demonstrated that many signaling pathways are involved in the regulation of EMT induced during fibrosis (Gonzalez and Medici, 2014). Recently, several signaling pathways have been regarded as important regulators of EMT in IPF. These include phosphoinositide 3-kinase (PI3K)/AKT-, Wnt-, transforming growth factor-beta (TGF-β)- and vascular endothelial growth factor (VEGF)-dependent pathways (Yan et al., 2014). Hence, elucidation of the underlying molecular mechanism may ultimately be beneficial for obtaining innovative therapeutic strategies against IPF.

Human differentiated embryonic chondrocyte expressed gene 1 (DEC1), also known as BHLHE40/Stra13/Sharp2, belongs to a structurally distinct class of basic helix- loop-helix (bHLH) proteins (Sato et al., 2016). It has been considered as a signaling mediator of diverse physiological processes including circadian rhythmicity, immunity, proliferation, apoptosis, senescence, tumorigenesis, and fibrosis (Honma et al., 2002; Qian et al., 2008; Gallo et al., 2018; Jia et al., 2018; Jarjour et al., 2019; Le et al., 2019; Li et al., 2019). A previous study has reported that *Dec1* deficiency suppressed cardiac perivascular fibrosis in hypertrophic hearts induced by transverse aortic constriction (TAC), resulting in preserved cardiac function (Le et al., 2019). However, the significance of DEC1 in PF has never been investigated.

In this study, we aimed to elucidate the effects of DEC1 on bleomycin (BLM)-induced PF and EMT in mice, and on TGF-β1-induced EMT in A549 cells. Furthermore, we explored the underlying downstream signaling mechanisms regulated by DEC1 *in vivo* and *in vitro*.

## 2 Materials and Methods

### 2.1 Reagents

Bleomycin was purchased from HANHUI PHARMACEUTICALS CO., LTD. (Zhejiang, China). Recombinant human TGF-β1 (Cat. No.: 100-21-2) was obtained from Peprotech (Rocky Hill, NJ, United States). The PI3K/AKT and Wnt/β-catenin pathway inhibitors, LY294002 (Cat. No.: HY-10108) and XAV-939 (Cat. No.: HY-15147), respectively, were obtained from MedChem Express (Monmouth Junction, NJ, United States). Dimethyl sulfoxide (DMSO) was purchased from Sigma–Aldrich (St. Louis, MO, United States). All other solvents used in this study were of an analytical grade or higher and acquired from commercial sources.

### 2.2 Human Samples

Lung tissues from IPF patients (*n* = 3) were collected in Zhongnan Hospital of Wuhan University. Three normal lung tissues from resection of cancer were used as control. Patients were diagnosed with IPF according to the American Thoracic Society (ATS)/European Respiratory Society (ERS) consensus diagnostic criteria (Raghu et al., 2018).

### 2.3 Gene Expression Omnibus (GEO) Database Analysis

Search for the keyword “IPF” in the GEO database, the website is https://www.ncbi.nlm.nih.gov/geo/. Identify the biochip data related to IPF, compare the IPF in the chip data with normal lung tissue, and analyze the differential expression of DEC1 between IPF and normal lung tissue. This study uses two gene chips (GSE53845 and GSE5774), of which the GSE53845 chip belongs to GPL6480 (Agilent-014850 Whole Human Genome Microarray 4 × 44K G4112F), including 40 cases of IPF and 8 cases of normal human lung tissue samples; GSE5774 chip belongs to GPL4225 (NIH- NIEHS/Agilent Human Familial IIP 43K array), including 26 cases of IIP and 9 cases of normal human lung tissue samples.

### 2.4 Animal Experiments

#### 2.4 1 Experimental Animals

*Dec1* ^+/−^ (C57BL/6 background) male and female mice were acquired from Prof. Yang Jian (Nanjing Medical University, Nanjing, China), whose *Dec1*-knockout (*Dec1* ^−/−^) mice (RBRC04841) were originally obtained from RIKEN BioResource Center. *Dec1* ^+/−^ males and females were mated to obtain male WT (*Dec1* ^+/+^) and KO (*Dec1* ^−/−^) littermates for experiments. All healthy male mice, weighing 20–26 g (9–10 weeks old) were kept under pathogen-free conditions on a 12-h light/dark cycle, at a room temperature of 25 ± 2°C and a relative humidity of 55 ± 5%. Before the PF model was established, the mice underwent an acclimatization period of at least 1 week.

#### 2.4 2 Murine Model of Pulmonary Fibrosis

To induce PF, mice were anesthetized with pentobarbital (6 ml/kg 1% pentobarbital) and treated once with 50 µL of 2.5 mg/kg BLM via intratracheal instillation on day 0. Mice in the control group received an equal volume of sterile saline.

#### 2.4 3 Animal Experimental Groups

1) To investigate the effect of DEC1 on PF, EMT and the activity of the PI3K/AKT/GSK-3β/β-catenin integrated signaling pathway, mice were randomly divided into four groups: WT + control group, WT + BLM group, KO + control group and KO + BLM group. BLM was dissolved in saline.
2) To verify the participation of the PI3K/AKT/GSK-3β/β-catenin integrated signaling pathway in PF, mice were randomly assigned into four groups: WT + BLM group, WT + BLM + DMSO group, WT + BLM + LY294002 group and WT + BLM + XAV-939 group. Mice began to receive the inhibitors (LY294002 and XAV-939) or DMSO *via* intraperitoneal injection 1 day before the BLM administration and LY294002 was administrated every other day for 21 days (25 mg/kg per injection) and XAV-939 was injected once daily for 11 days (10 mg/kg per injection). DMSO (10%) diluted in saline was used as a control and to dissolve the inhibitors (LY294002 and XAV-939).

On day 21 after the BLM or 0.9% saline intervention, the mice were anesthetized with 1% pentobarbital sodium or sacrificed under CO2, and were then subjected to bronchoalveolar lavage, and finally the lung tissues were rapidly collected. The left lung tissues were fixed in 4% paraformaldehyde for histological analysis, and the right lung tissues were stored at –80°C until being used for the hydroxyproline assay, quantitative real-time PCR (qRT-PCR) and western blotting.

### 2.5 Cell Culture and Intervention

A549 cell line were purchased from the Type Culture Collection of the Chinese Academy of Sciences (Shanghai, China) and cultured in RPMI-1640 medium (Hyclone, UT, United States) supplemented with 10% fetal bovine serum (FBS) (Gibco, Waltham, MA, United States) and 1% antibiotics (100 μg/ml streptomycin and 100 μg/ml penicillin) at 37°C in a 5% CO_2_ incubator.

To induce the EMT model *in vitro*, cells at 50–60% confluence were starved with serum-free medium for 12 h, and then subjected to recombinant human TGF-β1 (10 ng/ml) for 48 h. Cells were assigned into different groups as follows:

1) control group and TGF-β1 group.
2) shRNA-NC group, shRNA-*DEC1* group, TGF-β1 + shRNA-NC group and TGF-β1 + shRNA-*DEC1* group.
3) Vector group, *DEC1* cDNA group, *DEC1* cDNA + LY294002 group and *DEC1* cDNA + XAV-939 group. A549 cells were treated with LY294002 or XAV-939 for 1 h prior to the transfection with *DEC1* plasmids. LY294002 and XAV-939 were dissolved in 0.1% DMSO and diluted to 10 μM during cell treatment.

### 2.6 Cell Transfections

Lentiviruses containing short hairpin RNAs specially targeting *DEC1* (shRNA-*DEC1*) or the scramble control short hairpin RNA (shRNA-NC) were purchased from GeneChem (Shanghai, China). Cells were cultured for 24 h in a 6-well plate (8 × 10^4^ cells/well), and then transfected with scrambled shRNA or DEC1 shRNA at a MOI of 40. After 8 h, the medium was replaced with 10% RPMI-1640 medium for 72 h when the cells reached 70–80% confluence. Then the cells were incubated with 3 μg/ml puromycin to select stable cell line. Knockdown efficiency was verified by western blotting. For *DEC1* overexpression plasmids construction and transfection, human *DEC1* cDNA was purchased from GeneChem (Shanghai, China). Transfection was carried out using the Lipo8000™ Transfection Reagent (Beyotime, Shanghai, China) according to the manufacturer’s instructions. Overexpression efficiency was verified by western blotting.

### 2.7 Quantitative Real-Time PCR (qRΤ-PCR)

Total RNA was isolated using TRIzol (Invitrogen, Carlsbad, CA, United States) according to the manufacturer’s instructions. RNA reverse transcription was conducted using ReverTra Ace qPCR RT Kit (TOYOBO, Osaka, Japan) and qPCR was performed using UltraSYBR Mixture (CWBIO, Beijing, China). The relative mRNA expression levels were measured on the basis of the Ct value and relative to the endogenous reference gene, *GAPDH*, in accordance with the 2 ^−ΔΔCt^ method.

The mouse primer sequences used in the study were listed as follows: DEC1: forward, 5′-CGT​TGA​AGC​ACG​TGA​AAG​CA-3′, reverse, 5′-AAGTACCTC ACGGGCACAAG-3′; E-cadherin: forward, 5′-GACCG GAAGTGACTCGAAATG- 3′, reverse, 5′-CCC​TCG​TAA​TCG​AAC​AC CAAC-3′; Vimentin: forward, 5′-GCAGT ATGAAAGCGTGGCTG-3′, reverse, 5′-GCT​CCA​GGG​ACT​CGT​TAG​TG-3′; α- SMA: forward, 5′-GGA​CGT​ACA​ACT​GGT​ATT​GTG​C-3′, reverse, 5′-TCGGCAGTAGTC ACGAAGGA-3′; COL1A1: forward, 5′-GAC​GGG​AGT​TTC​TCC​TCG​G G-3′, reverse, 5′-GGG​ACC​CTT​AGG​CCA​TTG​TG-3′; COL1A2: forward, 5′-GGGCAAAA GAGAAGGATTGGTC-3′, reverse, 5′-AGCC ACAAGTGGTGCGAAT-3′; MMP2: forward, 5′-ACCTGAACACTTTC TATGGCTG-3′, reverse, 5′-CTTCCGCATGGTC TCGATG-3′; GAPDH: forward, 5′-TGA​AGG​GTG​GAG​CCA​AAA​G-3′, reverse, 5′-AGTCTTC TGGGTGGCAGTGAT-3′.

The human primer sequences used in the study were listed as follows: DEC1: forward, 5′-ATC​CAG​CGG​ACT​TTC​GCT​C-3′, reverse, 5′-TAAT TGCGCCG ATCCTTTCTC-3′; E-cadherin: forward, 5′- GAGAACGCA TTGCCACATACAC-3′, reverse, 5′-GCA​CCT​TCC​ATG​ACA​GAC​CC-3′; Vimentin: forward, 5′- AGTCCACT GAGTACCGGAGAC-3′, reverse, 5′- CAT​TTC​ACG​CAT​CTG​GCG​TTC-3′; GAPDH: forward, 5′-GTC​TCC​TCT​GAC​TTC​AAC​AGC​G-3′, reverse, 5′-ACCACCCTGTTG CTGTAGCCAA-3′.

### 2.8 Western Blotting

Proteins were extracted from lung tissues and A549 cells using RIPA lysis buffer (Sigma–Aldrich, St. Louis, MI, United States) supplemented with PMSF (Beyotime, Shanghai, China) and Phosphatase Inhibitor Cocktail (CWBIO). Tissue and cell lysates were centrifuged at 12,000 × *g* for 15 min at 4°C, and then the supernatants were immediately collected. Equal protein quantities were then subjected to 10% SDS-PAGE, transferred onto poly-vinylidene fluoride membranes (Millipore, Bedford, MA, United States), blocked with 5% skim milk for 1 h at room temperature, and then incubated with the indicated primary antibodies at 4°C overnight. The primary antibodies were as follows: DEC1 (NB100-1800SS, Novus, Centennial, CO, United States), E-cadherin (3,195, CST, Danvers, MA, United States), N-cadherin (13,116, CST), Vimentin (5,741, CST), AKT1/2/3 (ab179463, Abcam, Cambridge, MA, United States), p-AKT (Ser473) (4,060, CST), GSK-3β (ab32391, Abcam), p-GSK-3β (Ser9) (5,558, CST), β-catenin (ab32572, Abcam) and GAPDH (AS1039, Aspen, Wuhan, China). The membranes were then incubated with horseradish peroxidase-conjugated anti-rabbit secondary antibodies for 1 h at room temperature. Subsequently, the specific protein bands were visualized with an enhanced ECL kit (Thermo Fisher Scientific, Waltham, MA, United States), and then exposed to the electrochemiluminescence (ECL) system (Tanon, Shanghai, China).

### 2.9 Determination of Lung Hydroxyproline Level

To measure the total collagen content of the left lung, the hydroxyproline contents of lung tissues on day 21 after BLM administration were measured using the Hydroxyproline assay kit (Cat. No.: A030-2, Nanjing Jiancheng Bioengineering Institute, Nanjing, China) in accordance with the manufacturer’s method.

### 2.10 Histopathology Analysis

The lung samples were fixed with 4% formaldehyde, followed by de-hydration and embedding in paraffin. The tissues were cut into 3-μm-thick transverse sections. Then, hematoxylin and eosin (H&E) and Masson’s trichrome staining were performed using standard techniques. The degree of lung fibrosis was evaluated using the Ashcroft method (Ashcroft et al., 1988).

### 2.11 Immunofluorescence

For tissue Immunofluorescence (IF) staining, formalin-fixed and paraffin-embedded sections were deparaffinized in xylene, hydrated with an ethanol gradient and briefly washed with distilled water. Paraffin sections were placed in a repair box filled with EDTA antigen retrieval buffer (pH 8.0) and heated in a microwave oven for antigen retrieval. Next, the sections were incubated with goat serum for 30 min, followed by incubation with primary antibodies at 4°C overnight. The next day, the sections were incubated with different fluorescein-conjugated secondary antibodies for 50 min at room temperature in the dark, and nuclear staining with DAPI was performed for 10 min. Finally, images of IF staining were taken using a fluorescence microscope.

For cell IF staining, cells were plated and grown on sterilized glass coverslips. Cells were washed three times with cold PBS and fixed with 4% paraformaldehyde for 30 min, and then incubated with primary antibodies at 4°C overnight. Next, cells were incubated with fluorescein-labeled goat anti-rabbit secondary antibodies for 50 min at 37°C in the dark. Nuclear staining with DAPI was performed for 10 min. The cells were observed under a fluorescence microscope.

### 2.12 Statistical Analysis

All data in this study were analyzed with SPSS 21.0 or GraphPad Prism 8.0 and were presented as means ± SEM of at least three independent experiments. Student’s *t*-test and one-way ANOVA were used for comparison between two or multiple groups. *p* < 0.05 was considered statistically significant.

## 3 Results

### 3.1 DEC1 Is Increased in Idiopathic Pulmonary Fibrosis Patients and Bleomycin-Stimulated Pulmonary Fibrosis in Mice

**FIGURE 1:**
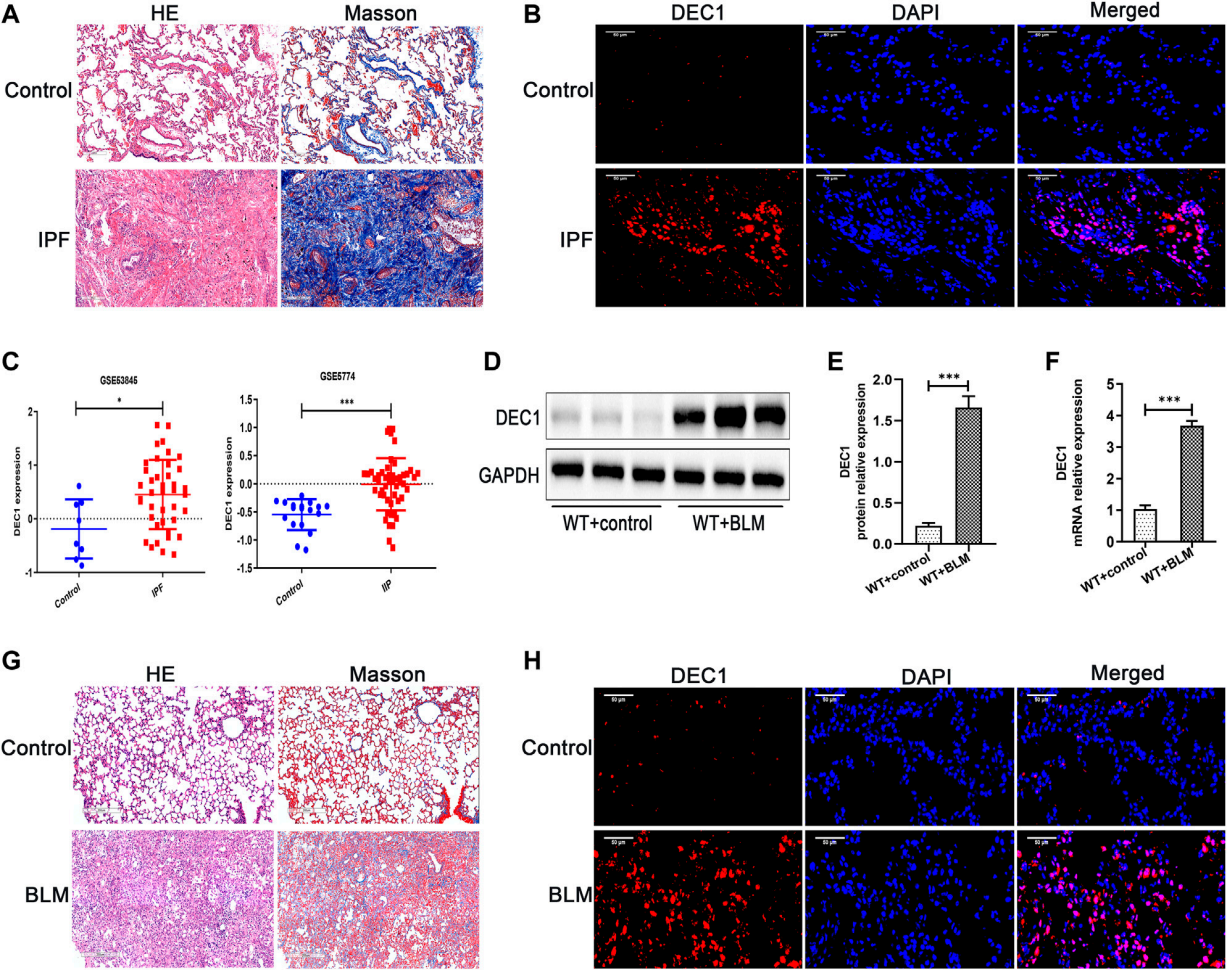
DEC1 is increased in IPF patients and BLM-stimulated PF in mice. **(A)** Representative HE staining and Masson’s trichrome staining of lung tissues from normal people and IPF patients. Scale bar = 200 μm *n* = 3. **(B)** Representative immunofluorescence staining of DEC1 (red) in the lung sections from normal people and IPF patients. Nuclei are stained with DAPI (blue). Scale bar = 50 μm *n* = 3. **(C)** The expression of DEC1 in the lung tissues from healthy people, IPF patients and IIP patients by analyzing GEO database. **(D)** The protein expression of DEC1 in the lung tissues were detected by western blotting. *n* = 6. **(E)** Statistical analysis of relative expression levels of proteins in **(D)**. **(F)** The mRNA expression of *DEC1* in the lung tissues were measured by qRT-PCR. *n* = 6. **(G)** Representative HE staining and Masson’s trichrome staining of lung tissues in mice. Scale bar = 200 μm *n* = 6. **(H)** Representative immunofluorescence staining of DEC1 (red) in the lung tissues of mice. Nuclei are stained with DAPI (blue). Scale bar = 50 μm *n* = 6. All data are shown as the mean ± SEM. Statistical analysis was performed by student’s t-test. *p < 0.05, **p < 0.01, ***p < 0.001, ^ns^ *p* > 0.05.

Before we study the functional role of DEC1 in PF, we first measured the expression of DEC1 in lung tissues from IPF patients. Just as we expected, DEC1 expression was obviously increased in the lungs from IPF (Figures 1A,B). Additionally, we found that DEC1 was highly expressed in IPF and IIP patients by analyzing data from GEO database (GSE53845 and GSE5774) (Figure 1C). To further address our assumption, we examined DEC1 expression in the BLM-induced PF murine model. We noticed that BLM significantly upregulated the protein and mRNA expression of DEC1 in lung tissues (Figures 1D–H). Consequently, these results verify that the expression of DEC1 is obviously enhanced in IPF patients and BLM-induced mice.

### 3.2 *Dec1* KO Ameliorates Bleomycin-Induced Pulmonary Fibrosis in Mice

**FIGURE 2:**
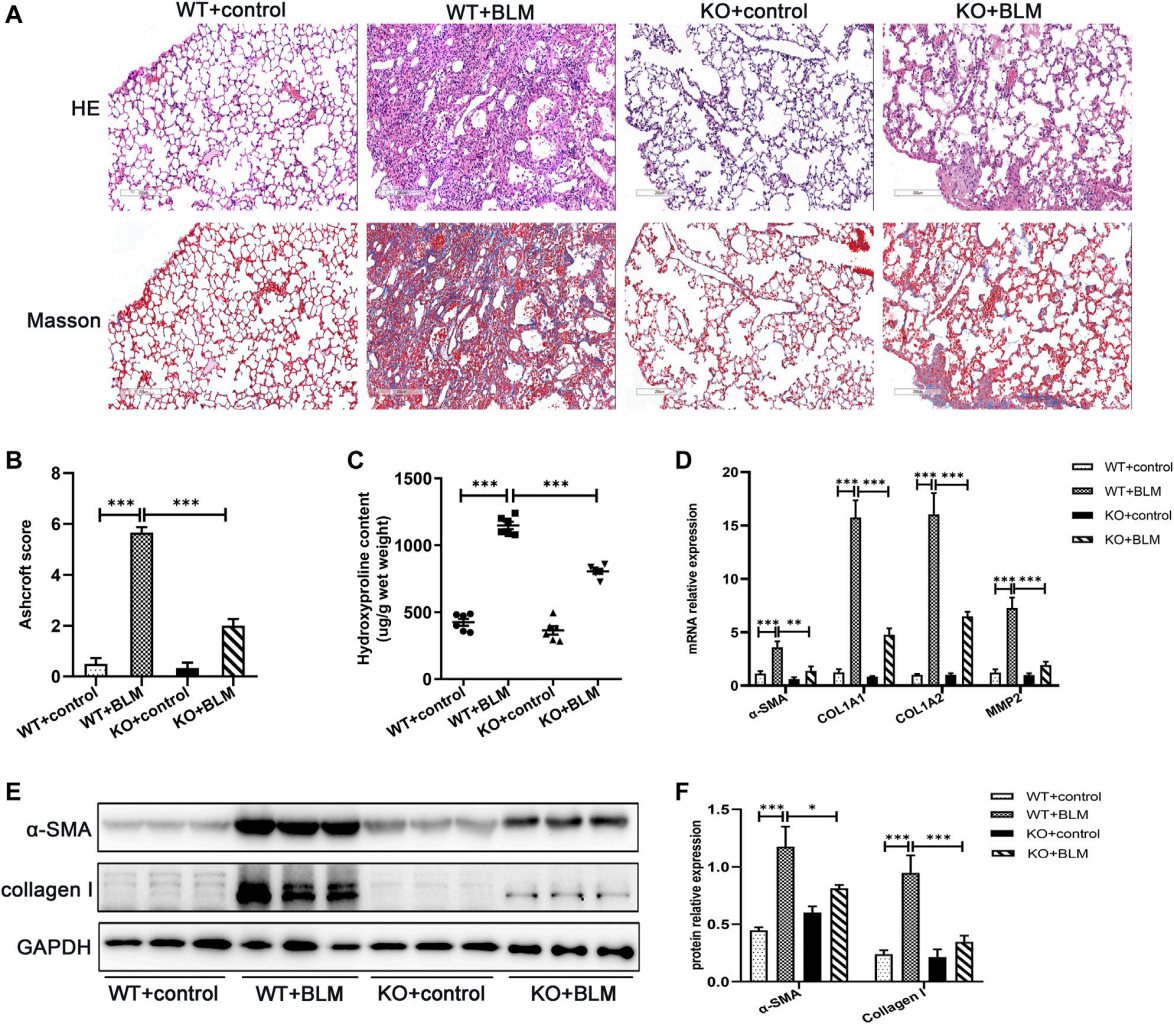
*Dec1* KO ameliorates BLM-induced PF in mice. WT and KO mice were treated with a single intratracheal injection of BLM (2.5 mg/kg) or saline for 21 days. **(A)** Representative HE staining and Masson’s trichrome staining of lung tissues in mice. Scale bar = 200 μm. **(B)** Ashcroft score was measured using the Ashcroft method. **(C)** Total collagen levels were assessed by measuring hydroxyproline content in the lung tissues of mice. **(D)** The mRNA expression of *α-SMA, COL1A1, COL1A* and *MMP2* in the lung tissues of mice were measured by qRT-PCR. **(E)** The protein expression of α-SMA and collagen I in the lung tissues of mice were detected by western blotting. **(F)** Statistical analysis of relative expression levels of proteins in E. *n* = 6. Data are shown as the mean ± SEM. Statistical analysis was performed by one-way ANOVA. *p < 0.05, **p < 0.01, ***p < 0.001, ^ns^ *p* > 0.05.

The role of DEC1 in PF induced by BLM was investigated using *Dec1* KO (*Dec1* ^−/−^) mice. The genotypes of the mice were identified by the protocol provided by Nanjing Medical University (Supplementary Figure S1A). After WT and KO mice were administrated with BLM or saline for 21 days, lung tissues were collected for analysis. Firstly, WT mice exposed to BLM demonstrated obvious deposition of collagen in the lungs compared with saline-treated WT mice, whereas the collagen content was clearly reduced in BLM-induced KO mice (Figures 2A,C). To quantify lung fibrosis, we evaluated the Ashcroft score in the lung sections images. As shown in Figure 2B, BLM-induced WT mice attained a higher Ashcroft score compared with saline-treated WT mice, whereas BLM-induced KO mice acquired lower scores compared with BLM-induced WT mice. Furthermore, we evaluated the mRNA expression of several fibrosis-related markers in the different groups, such as *α-SMA*, *COL1A1*, *COL1A2*, and *MMP2*. BLM significantly induced the expression of these fibrosis-related markers in WT mice; however, this induced expression was attenuated in the KO mice (Figure 2D). The protein levels of α-SMA and collagen I were altered in accordance with the mRNA results above (Figures 2E,F). Namely, *Dec1* KO suppressed the expression of fibrosis-related markers in the BLM-induced PF model. To sum up, DEC1 expression is increased in the BLM-induced PF model in mice and *Dec1* KO ameliorates the BLM-induced PF, suggesting that DEC1 is involved in the development of BLM-induced PF.

### 3.3 *Dec1* KO Inhibits EMT in the Bleomycin-Induced Pulmonary Fibrosis Murine Model

**FIGURE 3:**
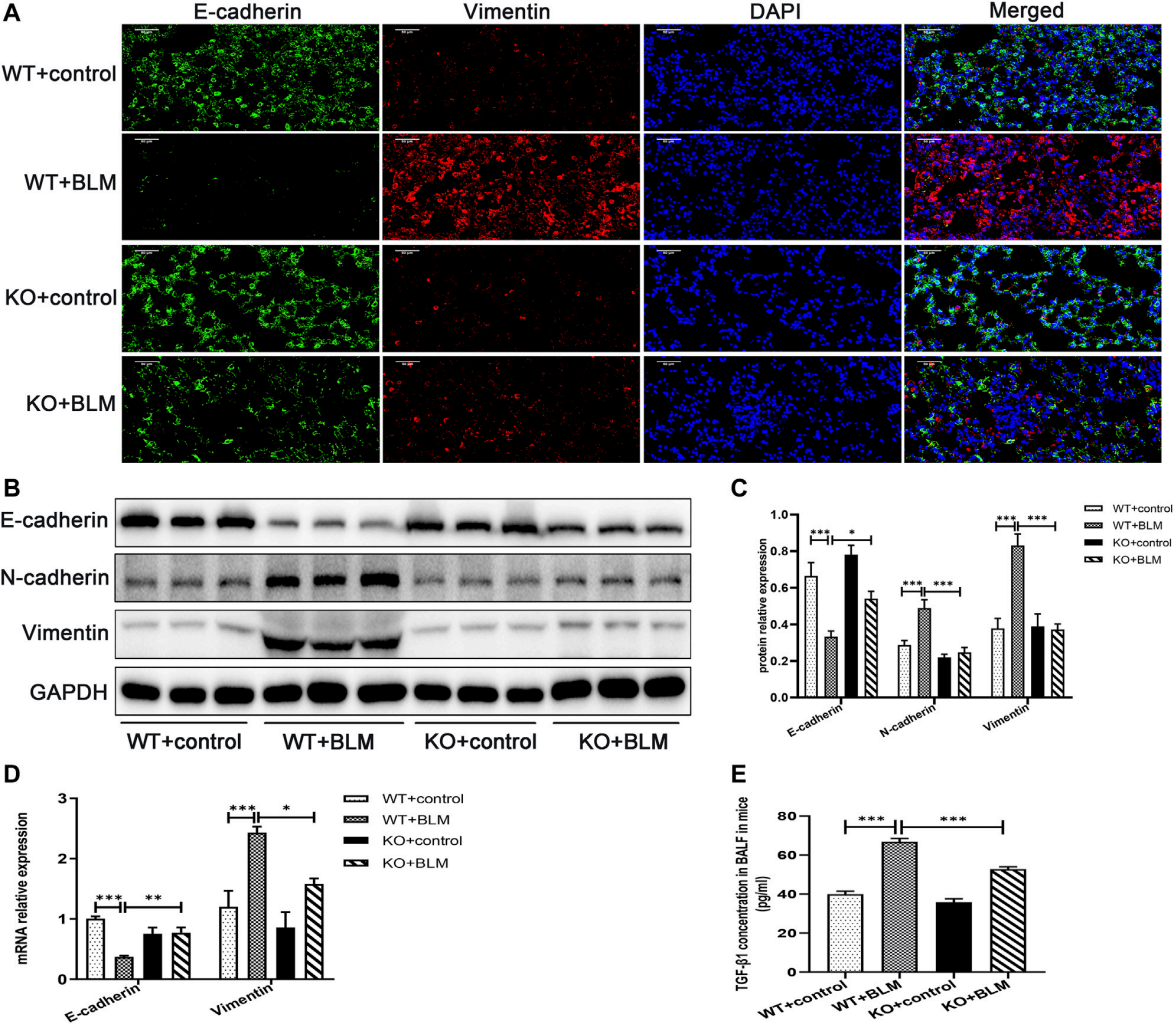
*Dec1* KO inhibits EMT in the BLM-induced PF murine model. WT and KO mice were treated with a single intratracheal injection of BLM (2.5 mg/kg) or saline for 21 days. **(A)** Dual immunofluorescent analysis for E-cadherin and Vimentin expression in the lungs tissue of mice. Scale bar = 50 μm. **(B)** The protein expression of E-cadherin, N-cadherin and Vimentin in the lung tissues of mice were measured by western blotting. **(C)** Statistical analysis of relative expression levels of proteins in **(B)**. **(D)** The mRNA expression of *E-cadherin* and *Vimentin* in the lung tissues of mice were assessed by qRT-PCR. **(E)** TGF-β1 levels in BALF of mice. *n* = 6. Data are shown as the mean ± SEM. Statistical analysis was performed by one-way ANOVA. *p < 0.05, **p < 0.01, ***p < 0.001, ^ns^ *p* > 0.05.

To investigate the mechanism involved in the development of PF *in vivo*, we examined the expression of the classic EMT-related markers, E-cadherin, N-cadherin and Vimentin, in the lung tissues of mice. EMT, the transition from an epithelial to a mesenchymal state, is one of the crucial processes in PF (Yang et al., 2019; Skibba et al., 2020). BLM induced the development of EMT in WT mice, which was associated with significant downregulation of the protein expression of the epithelial cell marker E-cadherin, and the upregulation of the protein level of the mesenchymal cell markers, N-cadherin and Vimentin. In contrast, the accumulation of E-cadherin was significantly increased and the expression of N-cadherin and Vimentin was obviously decreased in the BLM-induced *Dec1* KO mice compared with the BLM-induced WT mice (Figures 3A–C). Furthermore, the transcription levels of *E-cadherin* and *Vimentin* were regulated consistently with the corresponding protein results (Figure 3D). Additionally, as for TGF-β1 is a strong inducer of EMT, we made an examination of TGF-β1 in BALF, BLM significantly upregulated the concentration of TGF-β1 in mice BALF. However, TGF-β1 expression induced by BLM was attenuated in *Dec1*-knockout mice (Figure 3E). These results indicate that *Dec1* KO impedes the development of EMT in the BLM-induced PF model.

### 3.4 *Dec1* KO Suppresses the Activation of the PI3K/AKT/GSK-3β/β-Catenin Signaling Pathway in the Bleomycin-Induced Pulmonary Fibrosis Murine Model

**FIGURE 4:**
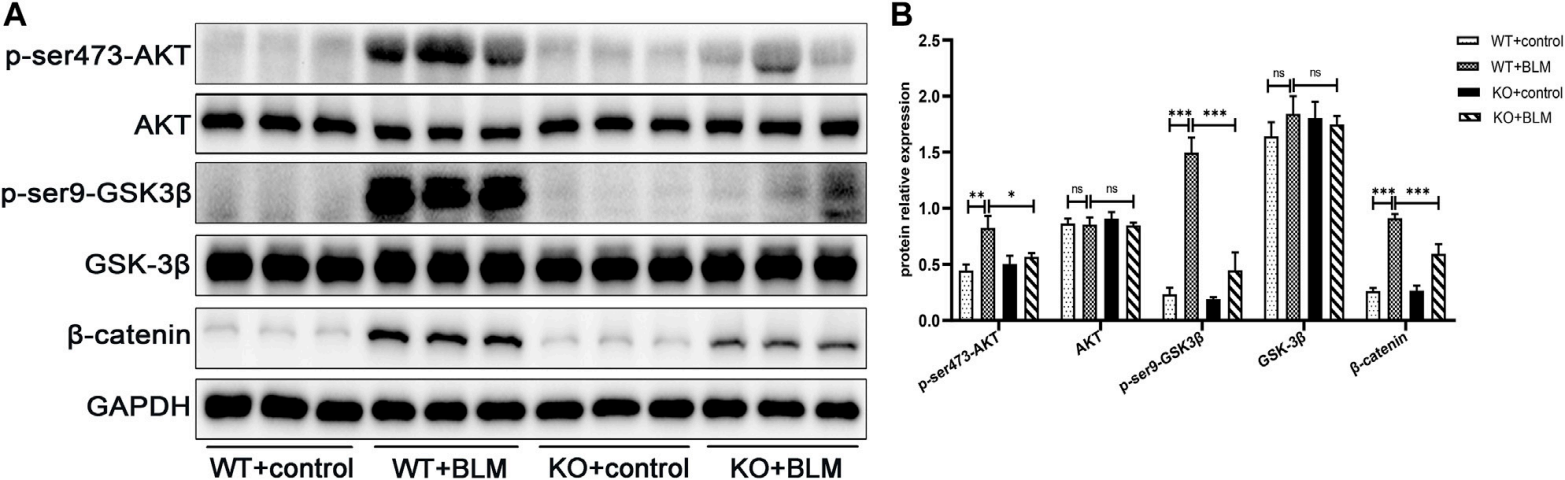
*Dec1* KO suppresses the activation of the PI3K/AKT/GSK-3β/β-catenin signaling pathway in the BLM-induced PF murine model. WT and KO mice were treated with a single intratracheal injection of BLM (2.5 mg/kg) or saline for 21 days **(A)** The protein expression of p-ser473-AKT, AKT, p-ser9-GSK3β, GSK-3β and β-catenin in the lung tissues of mice were detected by western blotting. **(B)** Statistical analysis of relative expression levels of proteins in A. *n* = 6. Data are shown as the mean ± SEM. Statistical analysis was performed by one-way ANOVA. *p < 0.05, **p < 0.01, ***p < 0.001, ^ns^ *p* > 0.05.

Next, we explored the specific signaling pathways involved in EMT during BLM-induced PF. To this end, we assessed the role of DEC1 in the PI3K/AKT/GSK-3β/β-catenin signaling pathway. We found that BLM upregulated AKT and GSK-3β phosphorylation and the level of β-catenin protein without obvious changes in the total AKT and GSK-3β expression levels in WT mice. In contrast, the levels of p-ser473-AKT, p-ser9-GSK3β and β-catenin were effectively decreased in BLM- induced KO mice, suggesting an involvement of the PI3K/AKT/GSK-3β/β-catenin signaling pathway in BLM-induced PF *in vivo* and that *Dec1* KO inhibits the activation of this BLM-induced pathway (Figures 4A,B).

### 3.5 Inhibition of the PI3K/AKT/GSK-3β/β-Catenin Signaling Pathway Relieved Bleomycin-Induced Pulmonary Fibrosis

**FIGURE 5:**
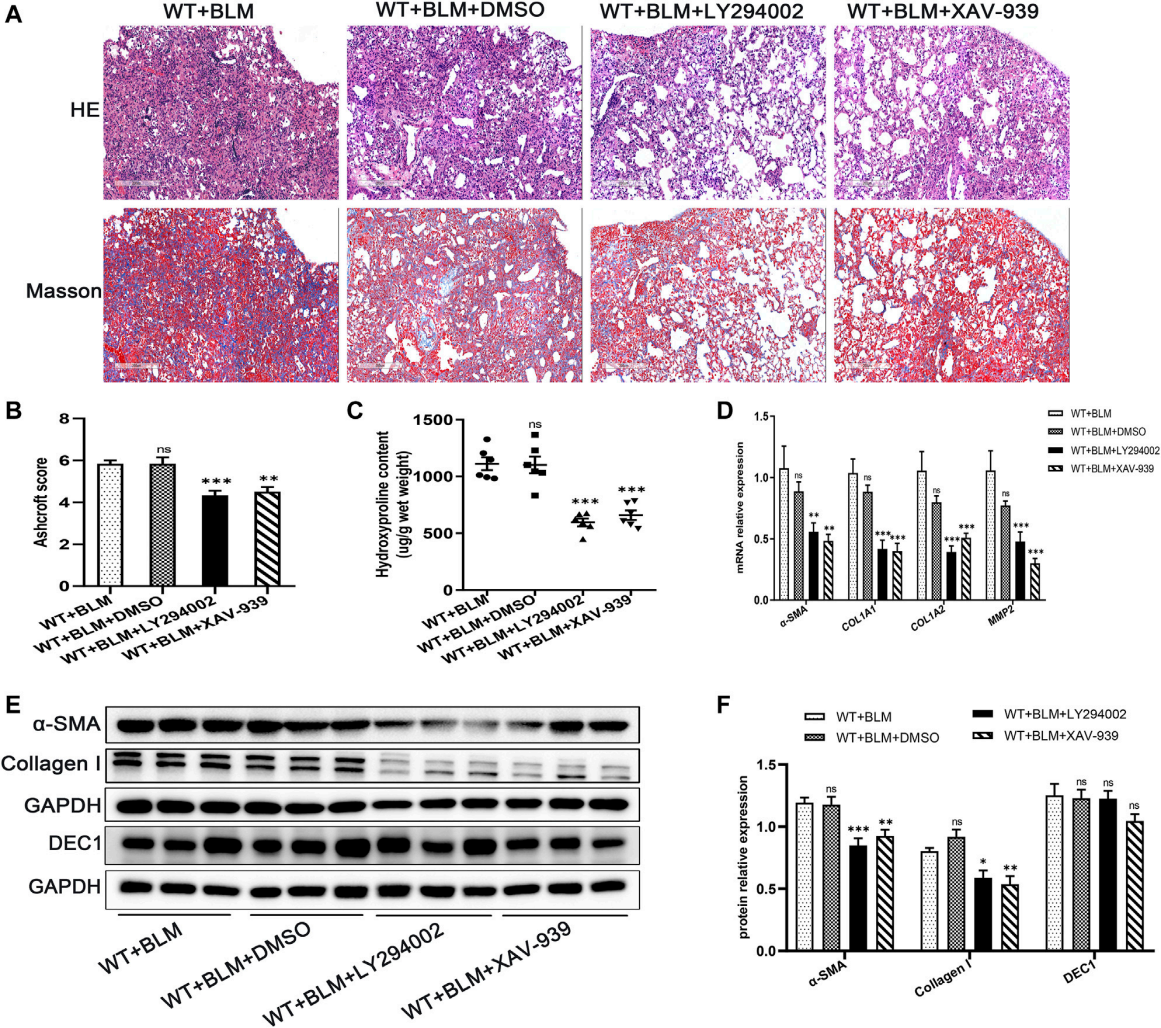
Inhibition of the PI3K/AKT/GSK-3β/β-catenin signaling pathway relieved BLM-induced PF. WT mice administrated by BLM were treated with or without LY294002 or XAV-939 or DMSO for 21 days. **(A)** Representative HE staining and Masson’s trichrome staining in the lung tissues in mice. Scale bar = 200 μm. **(B)** Ashcroft score was measured using the Ashcroft method. **(C)** Total collagen levels were assessed by measuring hydroxyproline content in the lung tissues of mice. **(D)** The mRNA expression of *α-SMA, COL1A1, COL1A*, and *MMP2* in the lung tissues of mice were measured by qRT-PCR. **(E)** The protein expression of α-SMA, collagen I and DEC1 in the lung tissues of mice were detected by western blotting. **(F)** Statistical analysis of relative expression levels of proteins in E. *n* = 6. Data are shown as the mean ± SEM. Statistical analysis was performed by one-way ANOVA. *p < 0.05, **p < 0.01, ***p < 0.001, ^ns^ *p* > 0.05.

To further clarify whether the PI3K/AKT/GSK-3β/β-catenin signaling pathway participates in EMT in the BLM-induced PF murine model, we examined the effect of LY294002 and XAV-939, potent inhibitors of the PI3K/AKT and Wnt/β-catenin signaling pathways, respectively, on lung fibrosis induced by BLM. Mice were administrated with LY294002, XAV-939 or DMSO. Interestingly, both LY294002 and XAV-939 ameliorated the degree of lung collagen deposition in the BLM-induced PF murine model (Figures 5A–C) and distinctly reversed the BLM-induced mRNA levels of the fibrosis-related markers, *α-SMA*, *COL1A1*, *COL1A2*, and *MMP2* (Figure 5D). Additionally, the changes in α-SMA and collagen I protein levels after intervening with LY294002 and XAV-939 were congruent with the mRNA results (Figures 5E,F). To further investigate the relationship between DEC1 and PI3K/AKT/GSK-3β/β-catenin signaling pathway, we detected the protein level of DEC1 under LY294002 and XAV-939 treatment. Importantly, both LY294002 and XAV-939 did not alter the BLM-induced protein level of DEC1 in lung tissues of mice (Figures 5E,F).

### 3.6 Inhibition of the PI3K/AKT/GSK-3β/β-Catenin Signaling Pathway Repressed Epithelial-Mesenchymal Transition in the Bleomycin-Induced Pulmonary Fibrosis Murine Model

**FIGURE 6:**
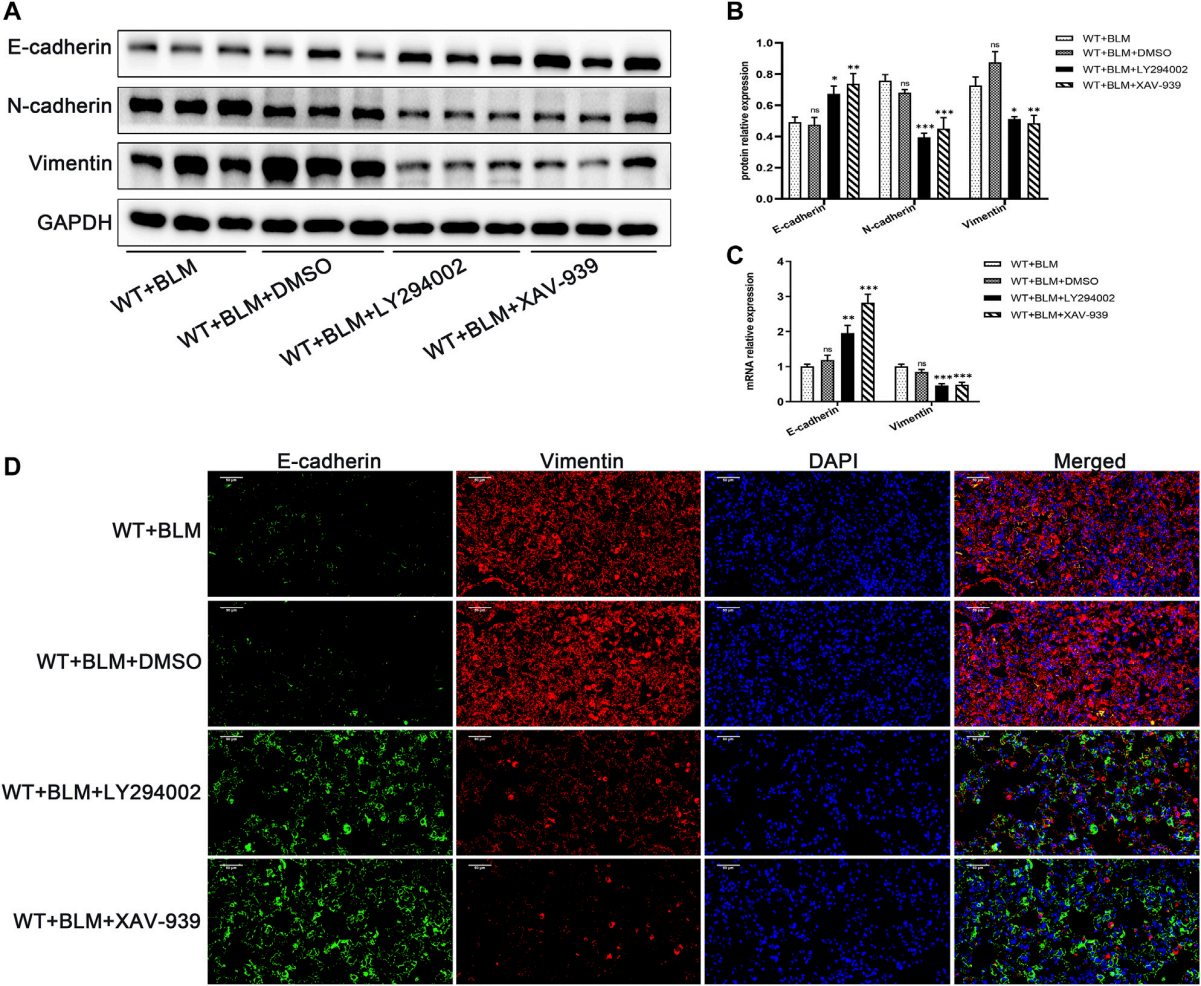
Inhibition of the PI3K/AKT/GSK-3β/β-catenin signaling pathway repressed EMT in the BLM-induced pulmonary fibrosis murine model. WT mice administrated by BLM were treated with or without LY294002 or XAV-939 or DMSO for 21 days. **(A)** The protein expression of E-cadherin, N-cadherin and Vimentin in the lung tissues of mice were measured by western blotting. **(B)** Statistical analysis of relative expression levels of proteins in A. **(C)** The mRNA expression of *E-cadherin* and *Vimentin* in the lung tissues of mice were assessed by qRT-PCR. **(D)** Dual immunofluorescent analysis for E-cadherin and Vimentin expression in the lungs tissue of mice. Scale bar = 50 μm *n* = 6. Data are shown as the mean ± SEM. Statistical analysis was performed by one-way ANOVA. *p < 0.05, **p < 0.01, ***p < 0.001, ^ns^ *p* > 0.05.

Subsequently, we examined whether LY294002 and XAV-939 affect the EMT process induced by BLM. Consistent with our expectation, LY294002 and XAV-939 clearly suppressed BLM-induced EMT, showing elevated protein level of E-cadherin and reduced protein levels of N-cadherin and Vimentin (Figures 6A,B). The changes in *E-cadherin* and *Vimentin* mRNA levels were consistent with the changes in their protein level (Figure 6C). Furthermore, dual immunofluorescence for E-cadherin and Vimentin was performed to confirm the changed expression in lung tissues following the intervention with the two inhibitors (Figure 6D). Taken together, these data demonstrate that DEC1 plays an important role in BLM-induced PF and EMT *via* the PI3K/AKT/GSK-3β/β-catenin signaling pathway.

### 3.7 DEC1 Expression is Upregulated in TGF-β1-Induced EMT in A549 Cells

**FIGURE 7:**
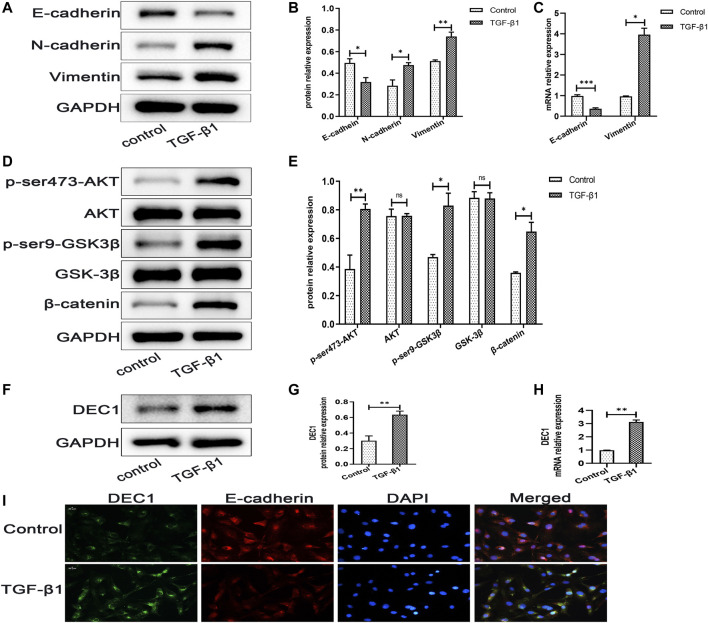
DEC1 expression is upregulated in TGF-β1-induced EMT in A549 cells. A549 cells were treated with TGF-β1 for 48 h. **(A)** The protein expression of E-cadherin, N-cadherin and Vimentin in A549 cells were measured by western blotting. **(B)** Statistical analysis of relative expression levels of proteins in A. **(C)** The mRNA expression of *E-cadherin* and *Vimentin* in A549 cells were assessed by qRT-PCR. **(D)** The protein expression of p-ser473-AKT, AKT, p-ser9-GSK3β, GSK-3β and β-catenin in A549 cells were measured by western blotting. **(E)** Statistical analysis of relative expression levels of proteins in D. **(F)** The protein expression of DEC1 in A549 cells were detected by western blotting. **(G)** Statistical analysis of relative expression level of proteins in F. **(H)** The mRNA expression of *DEC1* in A549 cells were detected by qRT-PCR. **(I)** Dual immunofluorescent analysis for DEC1 and E-cadherin expression in A549 cells. Scale bar = 20 μm *n* = 3. Data are shown as the mean ± SEM. Statistical analysis was performed by student’s t-test. *p < 0.05, **p < 0.01, ***p < 0.001, ^ns^ *p* > 0.05.

DEC1 has been reported to be involved in TGF-β1-induced EMT in PANC-1 cells (Wu et al., 2012). Additionally, our animal experiments demonstrated that *Dec1* KO ameliorated lung fibrosis by inhibiting EMT *in vivo*, hence, we examined the DEC1 expression in TGF-β1-induced A549 cells. Firstly, we observed that TGF-β1 successfully induced EMT. Compared with the control group, TGF-β1 downregulated the protein expression of E-cadherin, whereas it upregulated N-cadherin and Vimentin protein expression in A549 cells (Figures 7A,B). Furthermore, the mRNA levels of *E-cadherin* and *Vimentin* were altered similarly to the above protein results (Figure 7C). Secondly, TGF-β1 increased the protein expression levels of p-ser473-AKT and p-ser9-GSK3β and induced the accumulation of β-catenin without distinct changes in the total AKT and GSK-3β expression levels (Figures 7D,E), suggesting that TGF-β1 activated the PI3K/AKT/GSK-3β/β-catenin signaling pathway during the development of EMT. Thirdly, we assayed the DEC1 protein and mRNA expression levels in the two groups. An obviously higher level of DEC1 expression was found in the TGF-β1-induced group compared with the control group (Figures 7F–H). Additionally, dual immunofluorescence for E-cadherin and DEC1 confirmed that TGF-β1 downregulated the level of E-cadherin and upregulated DEC1 expression (Figure 7I).

### 3.8 *DEC1* KD Suppresses TGF-β1-Induced Epithelial-Mesenchymal Transition in A549 Cells

**FIGURE 8:**
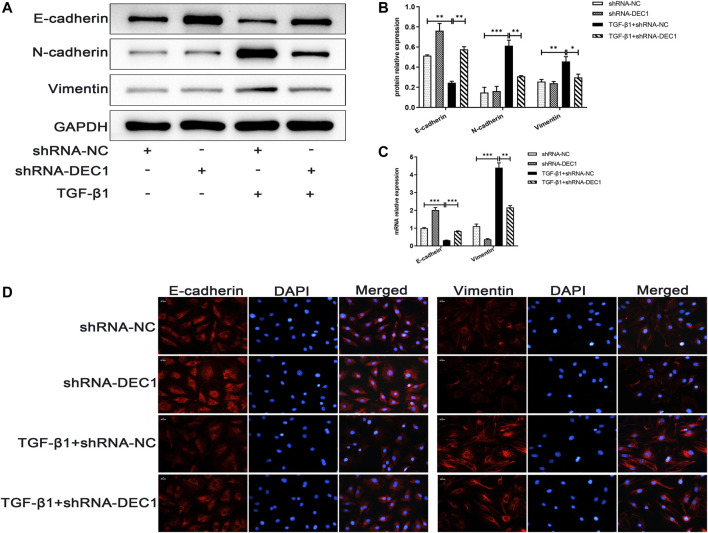
*DEC1* KD suppresses TGF-β1-induced EMT in A549 cells. shRNA-NC and shRNA-*DEC1* A549 cells were treated with or without TGF-β1 for 48 h. **(A)** The protein expression of E-cadherin, N-cadherin and Vimentin in A549 cells were measured by western blotting. **(B)** Statistical analysis of relative expression levels of proteins in A. **(C)** The mRNA expression of *E-cadherin* and *Vimentin* in A549 cells were assessed by qRT-PCR. **(D)** Immunofluorescent analysis for E-cadherin and Vimentin expression in A549 cells. Scale bar = 20 μm *n* = 3. Data are shown as the mean ± SEM. Statistical analysis was performed by one-way ANOVA. *p < 0.05, **p < 0.01, ***p < 0.001, ^ns^ *p* > 0.05.

Because DEC1 was significantly upregulated in TGF-β1-induced EMT in A549 cells, we further investigated whether DEC1 plays a significant role in the EMT process. We conducted experiments using the *DEC1* knockdown (KD) model, which was established by an shRNA lentiviral construct. The most effective shRNA, shRNA1-*DEC1*, was selected for the following experiments (Supplementary Figures S1B,S1C). We examined the changes in EMT-related markers in A549 cells after transfection with shRNA-*DEC1* lentiviruses. After TGF-β1 stimulation, shRNA-*DEC1* cells expressed higher protein level of E-cadherin and lower protein levels of N-cadherin and Vimentin compared with the shRNA-NC cells (Figures 8A,B). Additionally, the transcription levels of *E-cadherin* and *Vimentin* were changed in line with the protein results (Figure 8C). Moreover, the protein alterations in E-cadherin and Vimentin were once again confirmed by immunofluorescence (Figure 8D). Taken together, these results show that *DEC1* KD alleviates TGF-β1-induced EMT in A549 cells.

### 3.9 *DEC1* KD Reduces the Activation of the PI3K/AKT/GSK-3β/β-Catenin Signaling Pathway in A549 Cells

**FIGURE 9:**
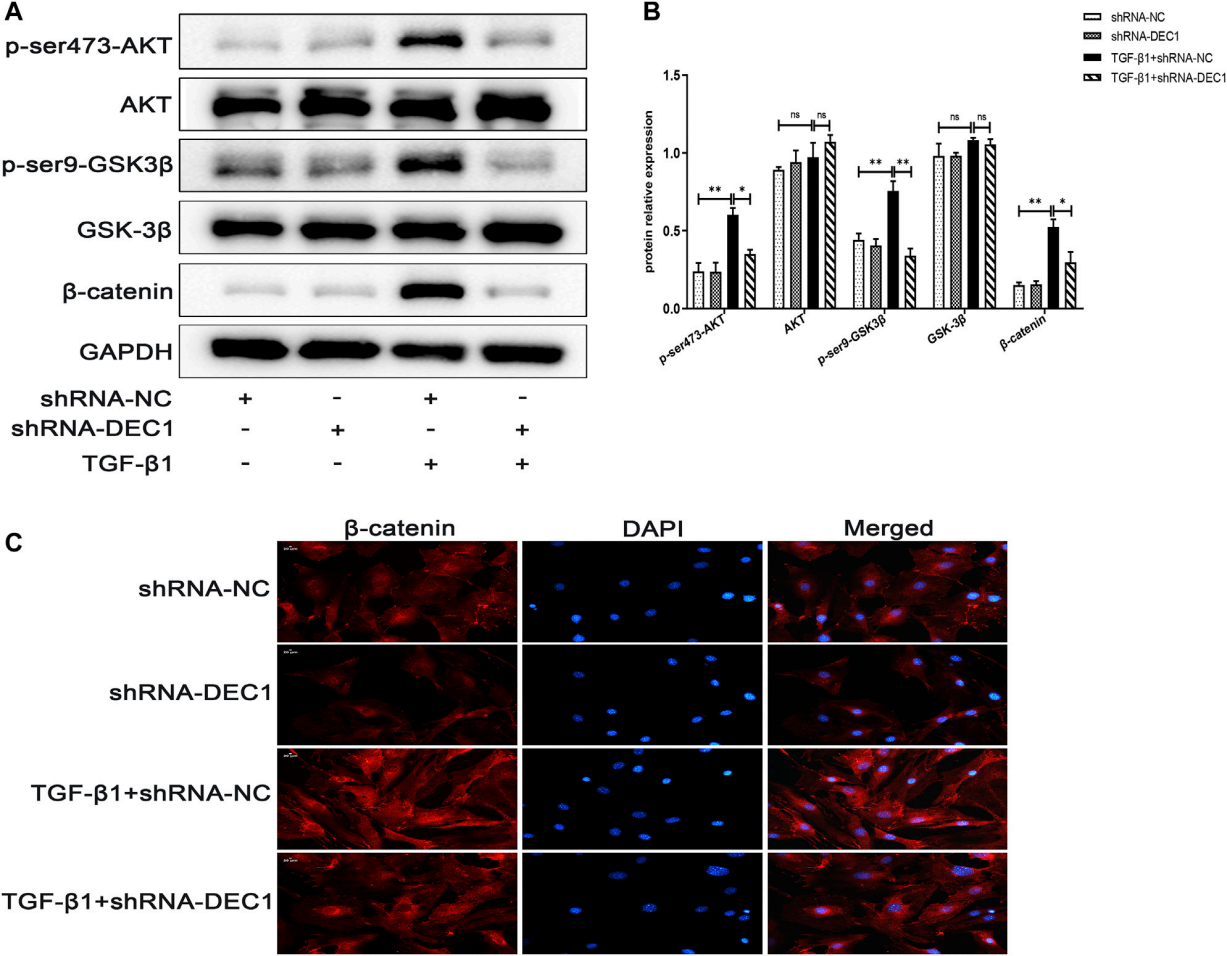
*DEC1* KD reduces the activation of the PI3K/AKT/GSK-3β/β-catenin signaling pathway in A549 cells**.** shRNA-NC and shRNA-DEC1 A549 cells were treated with or without TGF-β1 for 48 h. **(A)** The protein expression of p-ser473-AKT, AKT, p-ser9- GSK3β, GSK-3β, and β-catenin in A549 cells were measured by western blotting. **(B)** Statistical analysis of relative expression levels of proteins in A. **(C)** Immuno-fluorescent analysis for β-catenin expression in A549 cells. Scale bar = 20 μm *n* = 3. Data are shown as the mean ± SEM. Statistical analysis was performed by one-way ANOVA. *p < 0.05, **p < 0.01, ***p < 0.001, ^ns^ *p* > 0.05.

In order to determine the mechanism of action of DEC1 in TGF-β1-stimulated EMT, we analyzed the expression of PI3K/AKT/GSK-3β/β-catenin signaling pathway-related molecules in TGF-β1-stimulated EMT model. Interestingly, we discovered that a combination treatment of shRNA-*DEC1* and TGF-β1 clearly decreased the levels of p-ser473-AKT, p-ser9-GSK3β and β-catenin compared with TGF-β1-induced shRNA-NC cells (Figures 9A,B). Moreover, the protein alterations in β-catenin were certified by immunofluorescence (Figure 9C). Above results indicate that *DEC1* KD represses the TGF-β1-induced activation of the PI3K/AKT/GSK-3β/β-catenin signaling pathway in A549 cells.

### 3.10 *DEC1* Overexpression Induces Epithelial-Mesenchymal Transition and Activates the PI3K/AKT/GSK3β/β-Catenin Signaling Pathway in A549 Cells

**FIGURE 10:**
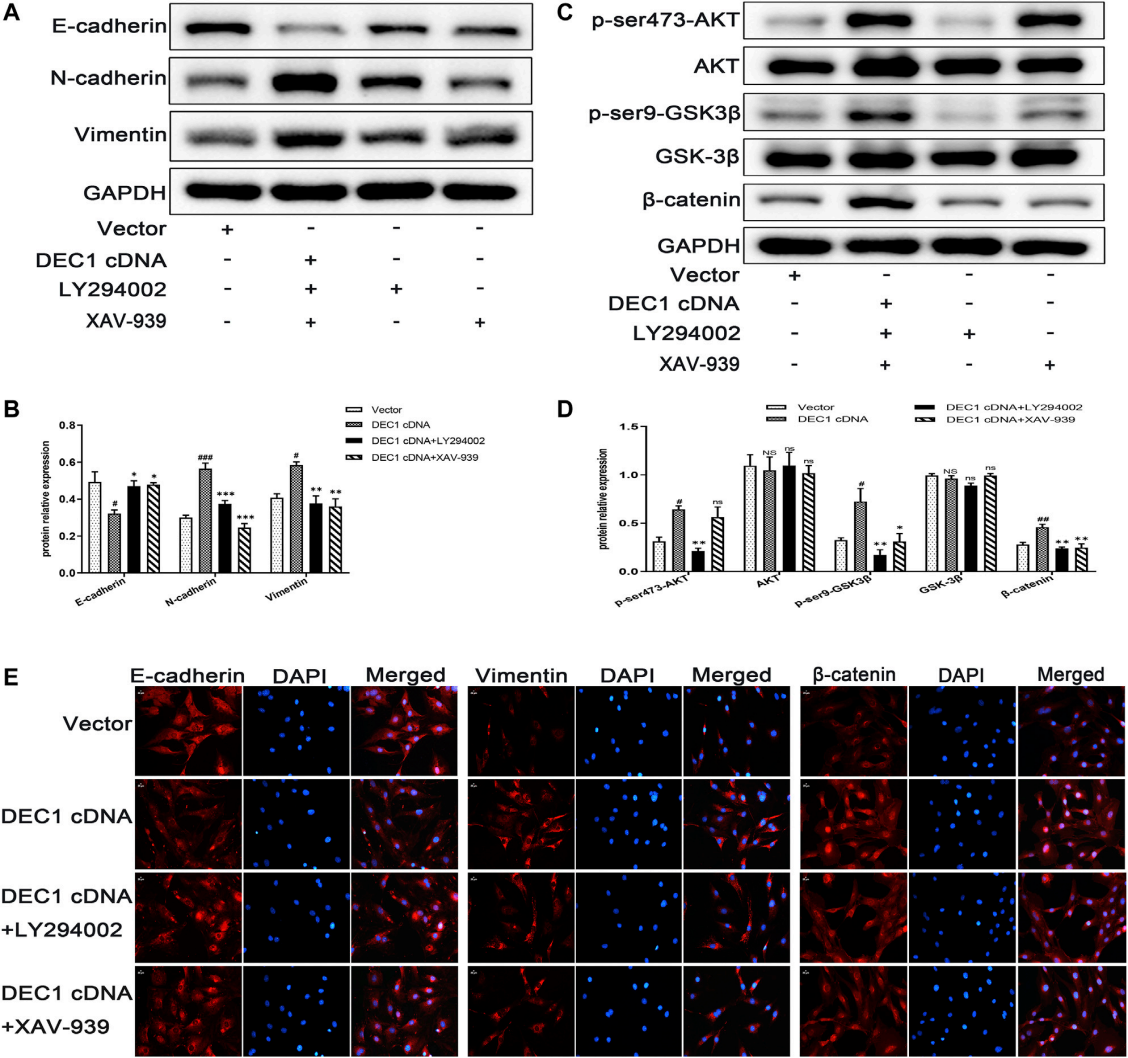
*DEC1* overexpression induces EMT and activates the PI3K/AKT/GSK3β/β-catenin signaling pathway in A549 cells. A549 cells were treated with LY294002 or XAV-939 for 1 h prior to the transfection with *DEC1* overexpression plasmids. **(A)** The protein expression of E-cadherin, N-cadherin and Vimentin in A549 cells were measured by western blotting. **(B)** Statistical analysis of relative expression levels of proteins in A. **(C)** The protein expression of p-ser473-AKT, AKT, p-ser9- GSK3β, GSK-3β, and β-catenin in A549 cells were measured by western blotting. **(D)** Statistical analysis of relative expression levels of proteins in C. **(E)**Immunofluorescent analysis for E-cadherin, Vimentin and β-catenin expression in A549 cells. Scale bar = 20 μm *n* = 3. Data are shown as the mean ± SEM. Statistical analysis was performed by one-way ANOVA. ^**#**^ *p* < 0.05, ^**##**^ *p* < 0.01 and ^NS^P>0.05 vs. Vector. *p < 0.05, **p < 0.01, ***p < 0.001 and ^ns^ *p* > 0.05 vs. *DEC1* cDNA.

To further verify the fibrogenic effect of DEC1 and the involvement of the PI3K/AKT/GSK-3β/β-catenin signaling pathway in EMT, we overexpressed DEC1 in A549 cells by *DEC1* plasmid and used the signaling pathway inhibitors, LY294002 and XAV-939. Overexpression plasmid successfully overexpressed DEC1 in A549 cells (Supplementary Figures S1D,S1E) and both inhibitors effectively reduced the phosphorylation levels of the corresponding proteins. More importantly, we observed that *DEC1* overexpression significantly reduced the protein expression of E-cadherin, whereas it enhanced the protein level of N-cadherin and Vimentin (Figures 10A,B). Moreover, *DEC1* overexpression distinctly increased the expression of p-ser473-AKT, p-ser9-GSK3β and β-catenin (Figures 10C,D). These results demonstrated that *DEC1* overexpression successfully induced EMT and activated the PI3K/AKT/GSK-3β/β-catenin signaling pathway. Meanwhile, cells were stimulated with inhibitors. Compared with the *DEC1* cDNA group, the expression of p-ser473-AKT, p-ser9-GSK3β and β-catenin was reduced by LY294002, and the expression of p-ser9-GSK3β and β-catenin was decreased by XAV-939. Furthermore, LY294002 and XAV-939 reversed the *DEC1* cDNA-induced EMT process, as evident from the increased protein levels of E-cadherin, whereas the distinctly decreased N-cadherin and Vimentin protein expression (Figures 10A,B). Additionally, the expression changes in E-cadherin, Vimentin and β-catenin in these groups were further validated by immunofluorescence (Figure 10E). The findings in this study demonstrated that *DEC1* overexpression induced EMT, and this effect was dependent on the PI3K/AKT/GSK-3β/β-catenin signaling pathway activation.

## 4 Discussion

IPF is a clinically common chronic fibrotic lung disease with unknown etiology, characterized by progressive PF, high disability rate and mortality (Richeldi et al., 2017). In this study, we demonstrated the role of DEC1 in the development of PF. The major findings of our study were that DEC1 was high expressed in IPF patients and BLM-challenged PF in mice. Moreover, *Dec1* KO markedly ameliorated PF in mice by inhibiting the EMT process in a BLM-induced PF model. Additionally, consistent with the *in vivo* results, *DEC1* KD suppressed TGF-β1-induced EMT, whereas *DEC1* overexpression induced EMT *in vitro*. Importantly, DEC1 regulated the activity of the PI3K/AKT/GSK-3β/β-catenin signaling pathway to regulate PF and the EMT process *in vivo* and *in vitro*. These results support the notion that DEC1 plays an important role in PF.

*Dec1* KO significantly attenuated BLM-induced PF. A single intratracheal dose of BLM in mice has been the most popular and best characterized animal model to investigate PF, which has provided valuable insight into the treatment of human IPF (Wollin et al., 2015; Glassberg et al., 2017). In previous studies, it has been shown that *Dec1* deficiency protects the heart from TAC-induced perivascular fibrosis through TGF-β1/pSmad3 and M1/M2 macrophage polarization (Le et al., 2019; Li et al., 2020). In this study, we evaluated whether DEC1 participates in the development of PF using *Dec1* KO mice. We found that *Dec1* KO distinctly alleviated BLM-induced PF. To the best of our knowledge, this is the first report showing that *Dec1* KO suppressed BLM-induced PF in mice. This observation implies that DEC1 plays a crucial role in development and progression of PF.

The precise molecular mechanisms by which DEC1 influences PF remain to be determined. We demonstrated that *Dec1* KO inhibited PF by impeding the EMT process *in vivo* and that *DEC1* KD repressed TGF-β1-induced EMT *in vitro*. In contrast, *DEC1* overexpression induced EMT *in vitro*. TGF-β1, a crucial mediator in tissue fibrosis, is a significant factor in promoting EMT and the development of PF (Akhurst and Hata, 2012; Aschner et al., 2020). We also found that *Dec1* KO alleviated BLM-induced upregulated expression of TGF-β1 in BALF in mice. The pathology of IPF has several characteristics including repetitive microscopic alveolar epithelial cell injury and dysregulated repair, unregulated proliferation and differentiation of fibroblasts into myofibroblasts, causing excessive ECM deposition, ultimately resulting in loss of parenchymal architecture and lung function (Borensztajn et al., 2013; Wollin et al., 2015). As reported previously, the origin of fibro-blasts and myofibroblasts in PF is still controversial; however, EMT in AEC2s is believed to be a significant source of the lung fibroblasts and myofibroblasts in PF (Willis et al., 2006; Chapman, 2011). Several reports have suggested that EMT is an important mechanism in the development of PF (Zhang et al., 2019; Chen et al., 2020; Yang et al., 2020). TGF-β1, one of the major profibrotic cytokines in IPF, is widely used to induce EMT in epithelial cells to explore the mechanism of PF (Qian et al., 2019; Kim et al., 2020). Accumulating evidence indicated that DEC1 regulates diverse biological processes involving EMT in cancer (Wu et al., 2012; Asanoma et al., 2015; Xiong et al., 2016). Therefore, we established a lung fibrosis model in mice and an EMT model in A549 cells using BLM and TGF-β1, respectively. Consistent with previous studies, our results showed that EMT developed in the BLM-induced PF model; however, it was alleviated when *Dec1* was knocked out in mice. *In vitro*, we obtained a similar trend, whereby TGF-β1 induced EMT in A549 cells and *DEC1* KD reversed the TGF-β1-stimulated EMT. Moreover, *DEC1* overexpression alone successfully induced EMT. These findings support the notion that DEC1 plays a crucial part in PF by regulating the EMT process.

The mechanism by which DEC1 affects the EMT process in PF is unclear. Another important finding in our study is that hyperactivation of the PI3K/AKT/GSK-3β/β-catenin signaling pathway was responsible for development of PF and EMT *in vivo* and *in vitro*. EMT is a complex process with the potential involvement of multiple signaling pathways (Lamouille et al., 2014). The PI3K/AKT signaling pathway, one of the most important signal transduction pathways in cells, is involved in the regulation of the EMT process (Wei et al., 2019). Moreover, GSK-3β is a downstream effector of the PI3K/AKT pathway, and AKT inhibits its activity by phosphorylating it at Ser9. GSK-3β also plays a crucial role in β-catenin phosphorylation and degradation, and the transfer of β-catenin into the nucleus, in which it forms a complex to activate target genes, such as E-cadherin, which is important in the development of EMT and fibrosis (Macdonald et al., 2009; Zheng et al., 2020). It has been documented that the PI3K/AKT signaling pathway and the Wnt/β-catenin signaling pathway are interconnected by the phosphorylation status of GSK-3β. The involvement of the PI3K/AKT/GSK-3β/β-catenin signaling pathway in EMT in gastric cancer cells has been reported and inhibition of this pathway suppresses EMT (Ge et al., 2018). However, the exact relationship between DEC1 and PI3K/AKT/GSK-3β/β-catenin signaling pathway has not been reported. A previous study has already showed that DEC1 deficiency led to a significant inhibition of PI3K/AKT/GSK3β signaling pathway. Additionally, LiCl, an agonist of Wnt/β-catenin signaling, could rescue the DA neuron loss of midbrain in the 6-month-old *Dec1* KO mice (Zhu et al., 2019). In this study, the PI3K/AKT/GSK-3β/β-catenin signaling pathway was clearly activated in BLM-treated mice, and in TGF-β1-stimulated and *DEC1* cDNA-induced cells. Furthermore, we found that DEC1 deficiency *in vivo* and *in vitro* inhibited the activity of the PI3K/AKT/GSK-3β/β-catenin signaling pathway. Interestingly, inhibition of the PI3K/AKT and Wnt/β-catenin signaling pathways by LY294002 and XAV-939, respectively, ameliorated the BLM-induced PF and EMT *in vivo*, and abated *DEC1* cDNA-induced EMT *in vitro*. Nevertheless, both LY294002 and XAV-939 did not alter the BLM-induced protein level of DEC1 in lung tissues of mice, suggesting PI3K/AKT/GSK-3β/β-catenin signaling pathway is a downstream of DEC1. Namely, DEC1 interacted with the PI3K/AKT and Wnt/β-catenin signaling pathways, leading to EMT, and ultimately giving rise to the development of PF.

DEC1 is a basic helix–loop–helix (BHLH) transcriptional factor. It is believed that DEC1 is a transcription factor that acts on a specific sequence. However, the transcription regulation mechanisms of DEC1 remain controversial (Jia et al., 2018), as one report suggested that DEC1 functions as a transcription activator through binding to the Sp1 element of its target genes (Li et al., 2006), whereas another report suggested it acts as a transcription repressor by directly binding to the E-box region of its target genes (Li et al., 2003). In addition, it has been documented that PI3K/AKT signaling interacts with Sp1 (Yan et al., 2015). Thus, in our study, DEC1 may promote or inhibit the expression of its target factors by acting on a specific sequence region, which is what we are going to investigate and confirm in the future.

In summary, the present study demonstrated two important findings. First, DEC1 exerted a considerable role in PF by regulating the EMT process. Second, DEC1 affected EMT by regulating the activity of the PI3K/AKT/GSK-3β/β-catenin signaling pathway. Thus, our findings give credence to the hypothesis that DEC1 is crucial in the pathogenesis of PF. These findings shed light on the better understanding of mechanisms regulating PF. Finally, our study indicates that DEC1 and the mechanism described above may be potential molecular therapeutic targets in IPF.

## Data Availability

The original contributions presented in the study are included in the article/Supplementary Material, further inquiries can be directed to the corresponding authors.

## Supplementary Material

Supplementary Figure S1
**(A)** The genotypes of the mice were detected by PCR. **(B)** The efficiency of DEC1 knockdown lentivirus containing short hairpin RNAs in A549 cells was assessed by western blotting. **(C)** Statistical analysis of relative expression level of DEC1 proteins in A549 cells in **(B)**. **(D)** The efficiency of DEC1 overexpression plasmids in A549 cells was assessed by western blotting. **(E)** Statistical analysis of relative expression level of DEC1 proteins in A549 cells in **(D)**. *n* = 3. Data are shown as the mean ± SEM. Statistical analysis was performed by one-way ANOVA. **p* < 0.05, ***p* < 0.01, ****p* < 0.001, ns *p* > 0.05.
