# Supplementary figures and images for: Dec1 Deficiency Ameliorates Pulmonary Fibrosis Through the PI3K/AKT/GSK-3β/β-Catenin Integrated Signaling Pathway
Source: Front Pharmacol. 2022 Mar 9;13:829673. doi: 10.3389/fphar.2022.829673 (PMC8959854; doi:10.3389/fphar.2022.829673)

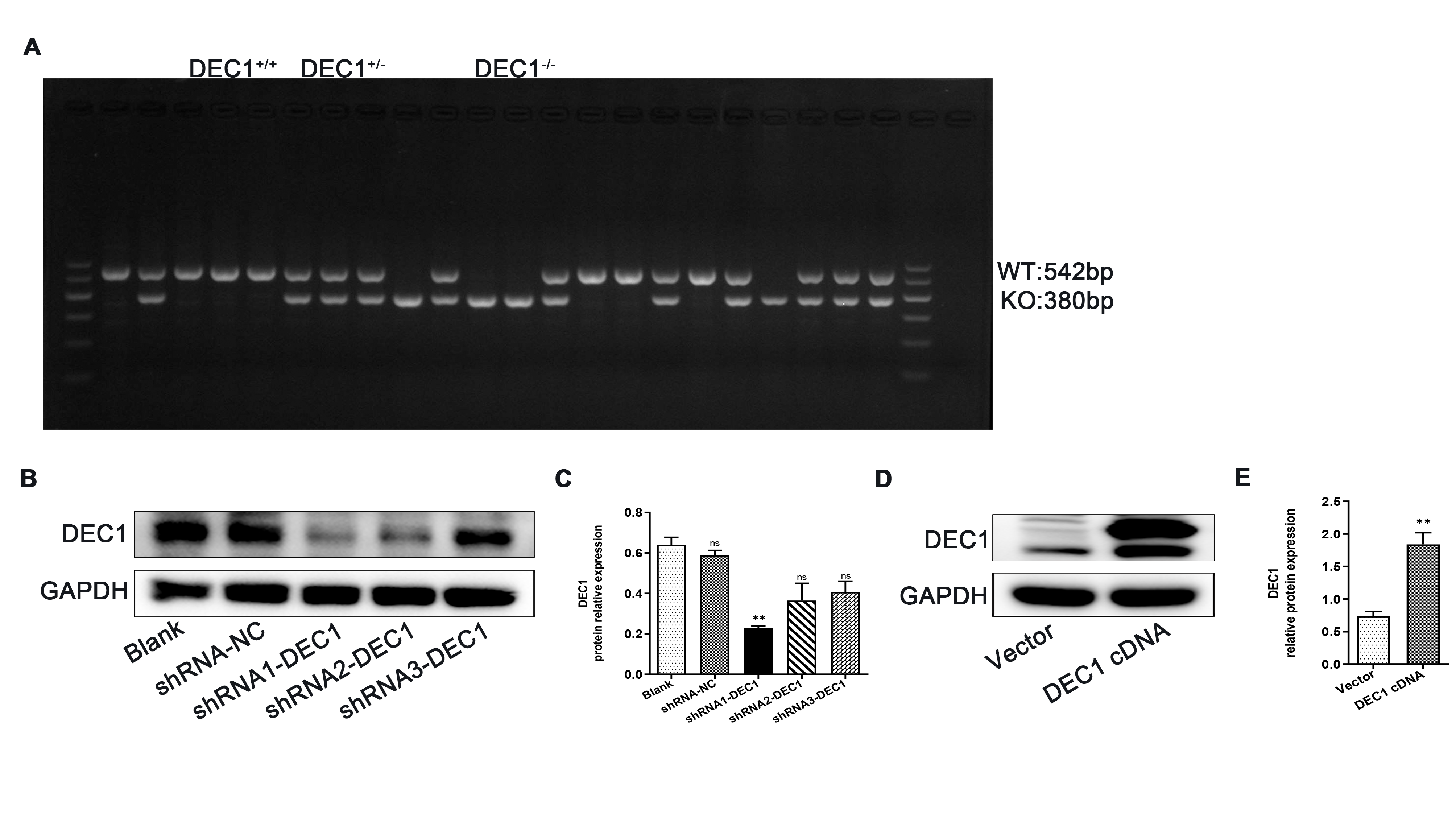

Supplement: Supplementary file 1 [file Image1.tif]
